# Increased Risk of Dementia in Patients with Mild Traumatic Brain Injury: A Nationwide Cohort Study

**DOI:** 10.1371/journal.pone.0062422

**Published:** 2013-05-01

**Authors:** Yi-Kung Lee, Sheng-Wen Hou, Ching-Chih Lee, Chen-Yang Hsu, Yung-Sung Huang, Yung-Cheng Su

**Affiliations:** 1 Emergency Department, Buddhist Tzu Chi Dalin General Hospital, Chiayi, Taiwan; 2 School of Medicine, Tzu Chi University, Hualien, Taiwan; 3 Emergency Department, Shin-Kong Wu Ho-Su Memorial Hospital, Taipei, Taiwan; 4 Community Medicine Research Center and Institute of Public Health, National Yang-Ming University, Taipei, Taiwan; 5 Department of Otolaryngology, Buddhist Dalin Tzu Chi General Hospital, Chiayi, Taiwan; 6 Cancer Center, Buddhist Dalin Tzu Chi General Hospital, Chiayi, Taiwan; 7 Department of Public Heath, National Taiwan University, Taipei, Taiwan; 8 Division of Neurology, Department of Internal Medicine, Buddhist Dalin Tzu Chi General Hospital, Chiayi, Taiwan; Baylor College of Medicine, United States of America

## Abstract

**Background:**

It is known that the risk of dementia in patients with moderate to severe traumatic brain injury (TBI) is higher. However, the relationship between mild traumatic brain injury (mTBI) and dementia has never been established.

**Objectives:**

We investigated the incidences of dementia among patients with mTBI in Taiwan to evaluate if there is higher risk compared with general population.

**Methods:**

We utilized a sampled National Health Insurance (NHI) claims data containing one million beneficiaries. We followed all adult beneficiaries from January 1, 2005 till December 31, 2009 to see if they had been diagnosed with dementia. We further identify patients with mTBI and compared their risk of dementia with the general population.

**Results:**

We identified 28551 patients with mTBI and 692382 without. After controlled for age, gender, urbanization level, socioeconomic status, diabetes, hypertension, coronary artery disease, hyperlipidemia, history of alcohol intoxication, history of ischemic stroke, history of intracranial hemorrhage and Charlson Comorbidity Index Score, the adjusted hazard ratio is 3.26 (95% Confidence interval, 2.69–3.94).

**Conclusions:**

TBI is an independent significant risk factor of developing dementia even in the mild type.

## Introduction

Dementia is a disorder defined by impairment of memory with at least one other cognitive deficit (aphasia, apraxia, agnosia, executive function) from previous level of function. [1] It is estimated that 35.6 million people worldwide suffered from Alzheimer’s disease (AD), the most common form of dementia, in 2010, and the costs associated with dementia were estimated to be 604 billion US dollars. Because of its progressive neurodegenerative nature, the burden of dementia will become increasingly significant in an aging population. [2].

Although the etiology is not well known, risk factors of dementia such as age, family histories and genetic factors have been extensively studied. Recently, traumatic brain injury (TBI) has been evoked as one possible precipitating factor.[3]–[8] Every year, about 1.4 million people suffer from TBI in the United States. TBI initiates many different signaling cascades throughout the brain that impact both pathophysiological and neuroprotective processes. Depending on the severity, survivors may suffer from a wide variety of symptoms such as neurological deficits, cognitive, behavioral and emotional impairments. [7] Although the link between TBI and development of dementia is complex, the axonal damage after TBI is also a key manifestation of AD and may be responsible for the development of dementia. [9].

A Systematic review has found that AD was associated with moderate and severe TBI, but not with mild TBI unless there was loss of consciousness, and the evidence for the latter was limited. [3] Our study utilized large-scale administrative data to explore the associations between patients with mild traumatic brain injury (mTBI) and dementia. Results of this study would provide clinicians with further insight on this frequently encountered situation.

## Methods

### Ethics Statement

This study was initiated after approved by the Institutional Review Board of Buddhist Dalin Tzu Chi General Hospital, Taiwan. Since all identifying personal information was stripped from the secondary files before analysis, the review board waived the requirement for written informed consent from the patients involved.

### Database

The National Health Insurance (NHI) program, which provides compulsory universal health insurance, was implemented in Taiwan in 1995. It enrolls up to 99% of the Taiwanese population and contracts with 97% of all medical providers. The database contains comprehensive information on insured subjects, including gender, date of birth, residential or work area, dates of clinical visits, the International Classification of Diseases (Ninth Revision) Clinical Modification (ICD-9-CM) codes of diagnoses, details of prescriptions, expenditure amounts and outcome at hospital discharge (recovered, died, or transferred out). A random sample with 1,000,000 people based on the 2005 reimbursement data was established for public access, and the group did not differ statistically significantly from the larger cohort in age, gender or health care costs, as reported by the Taiwan National Health Research Institute. [10], [11] The sampled group was used as our study cohort.

To avoid financial issues for patients with major illnesses, the NHI specifies 31 categories of catastrophic illness (e.g., dementia, cancers, chronic renal failure, etc.) that are exempt from co-payment. The attending physician of a patient diagnosed as catastrophic illness can submit related information in application for a catastrophic illness certificate (CIC). A committee formally reviews applications, and if approved, patients are then exempted from co-payment. [12].

### Study Population

**Figure 1:**
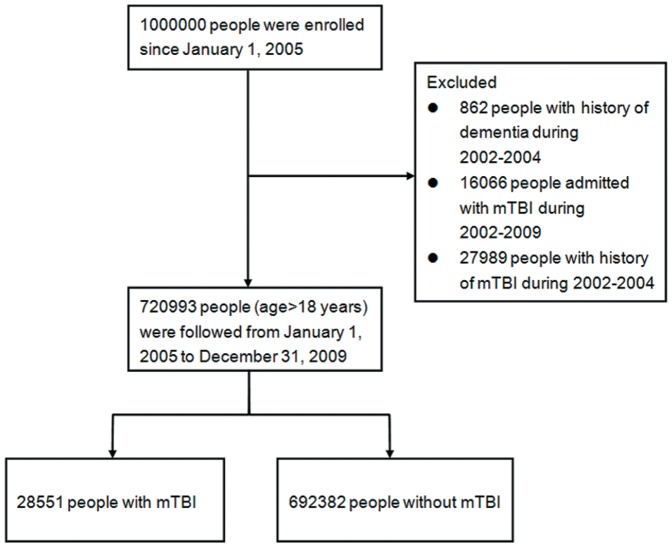
Flow diagram of the population-based study.

The sampled population has been followed from January 1, 2002 to December 31, 2009 (a total of eight years). First, We identified people older than 18 years who were still alive in 2005 as our study cohort. Mild traumatic brain injury (mTBI) was defined by ICD-9-CM code head concussion (850.0, 850.1, 850.5, or 850.9), intracranial injury of other and unspecified nature (854.0), or head injury, unspecified (959.01). [13] Dementia was defined by ICD-9-CM code 290 or 331.0 (Alzheimer’s disease) in any position of the diagnoses. To maximize case ascertainment, only 1) patients registered with dementia in the CIC or 2) those prescribed with either cholinesterase inhibitors (rivastigmine, donepezil and galantamine) or NMDA receptor antagonist (memantine) for dementia were included. Second, We excluded all patients with mTBI and dementia diagnosed before January 1, 2005. To ensure that exposed patients were not misclassified, we also excluded patients who had ever hospitalized with traumatic brain injuries to ensure the enrolled patients with mTBI were discharged directly after visits. After exclusion, we identified 28551 people with mTBI and 692382 without mTBI. Each person was tracked for 5 years from January 1, 2005 till December 31, 2009 to identify if he or she was diagnosed with dementia. These patients were then linked to the administrative data for the period 2005–2009 to calculate disease-free survival time, with cases censored for patients who drew back guarantees from the NHI Program or were still robust at end of follow-up (Figure 1).

### Covariates

To better understand the risk of mTBI on dementia, several covariates were included for analysis. First, patient demographics included age, gender, urbanization level (urban, suburban, and rural areas) and socio-economic status (SES). The insurance enrollee category was used as a proxy measure of SES. Second, prevalence of selected comorbid conditions (diabetes, hypertension, coronary artery disease, hyperlipidemia, history of alcohol intoxication, history of ischemic stroke and intracranial hemorrhage) and Charlson comorbidity index (CCI) were included. CCI is a scoring system that includes weighting factors on important concomitant diseases that has been validated for use with ICD-9-CM coded administrative databases. [14], [15].

### Statistical Analysis

The SAS statistical package, version 9.2 (SAS Institute, Inc., Cary, NC) was used for data analysis. Pearson’s chi-square test was used for categorical variables. The Nelson-Aalen cumulative hazard estimates were plotted to show different trends between patients with mTBI and those without. Cox proportional hazard regression model was then used to calculate the hazard ratio of dementia for people with mTBI after adjustments for age, gender, urbanization level, SES, diabetes, hypertension, coronary artery disease, hyperlipidemia, history of ischemic stroke, history of intracranial hemorrhage and CCI.

We did a subgroup analysis based on people older than 65 years to check if the effects of mTBI on developing dementia are similar. We also evaluate the risk of dementia in patients who had been hospitalized because of TBI to see if there is ‘dose-response’ effect in the relation of TBI and dementia.

To further assess the robustness of our results, we did sensitivity analyses [16] to evaluate how large the effect of an unmeasured confounder would be needed to account for the results. A two-tailed P value of <0.05 was considered significant.

## Results

**Figure 2:**
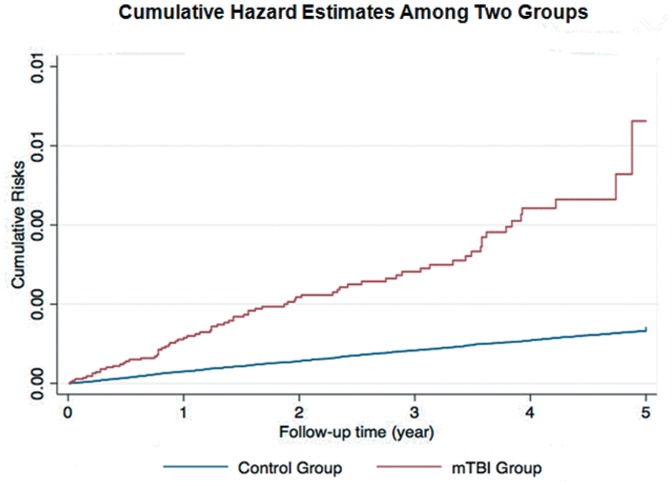
Nelson-Aalen plot showed higher cumulative risk of dementia in the mTBI group.

The distribution of demographic characteristics and selected morbidities is shown in Table 1. There were 28551 patients in the mTBI group and 692382 in the unexposed group. The total follow-up periods in the two groups were 70561 and 3348132 person-years, respectively. The percentage of computed tomography of examination in mTBI group is 13.7%. Patients with mTBI were significantly older. They were also more likely to have diabetes, hyperlipidemia, coronary artery disease, history of alcohol intoxication, ischemic stroke, intracranial hemorrhage, higher CCI and lower socioeconomic status. At the end of follow-up, 1071 patients had been confirmed for dementia, with 127 in the patients with mTBI and 944 in those without. The average duration from TBI to the diagnosis of dementia is 1.0 years (95% CI, 0.77–1.23). The incidence rate of dementia is 1.8 per 1000 person-year in patients with mTBI and 0.3 per 1000 person-year in those without. The crude hazard ratio (HR) of dementia between the two groups was 6.34 (95% Confidence interval [CI], 5.2–7.7), and the Nelson-Aalen plot showed higher cumulative risk in the mTBI group. (Figure 2).

**Table 1:** Baseline characteristics of the mTBI group and the unexposed group.

	mTBI Group (n = 28551)		Control Group (n = 692382)		
Variables	No.	%	No.	%	*P*-value
Gender					0.030
Male	13705	48.0	336897	48.7	
Patient age					<0.001
18–44 yrs	14766	51.7	402098	58.1	
45–54 yrs	4555	16.0	134834	19.5	
55–64 yrs	3202	11.2	71058	10.3	
65–74 yrs	2854	10.0	50657	7.3	
75 yrs and more	3174	11.1	33735	4.9	
Mean Age	45.9		43.1		
Charlson Comorbidity Index Score					<0.001
0	15201	53.2	419637	60.6	
1	7365	25.8	163851	23.7	
≥2	5985	21.0	108894	15.7	
Diabetes	3035	10.6	54672	7.9	<0.001
Hyperlipidemia	3345	11.7	71930	10.4	<0.001
Hypertension	5860	20.5	111459	16.1	<0.001
Coronary artery disease	2869	10.1	49241	7.1	<0.001
History of alcohol intoxication	504	1.8	5635	0.8	<0.001
Ischemic stroke	220	0.8	2334	0.3	<0.001
Intracranial hemorrhage	1871	6.6	26247	3.8	<0.001
Socioeconomic status					<0.001
Low	14631	51.3	303420	43.8	
Moderate	11299	39.6	275842	39.8	
High	2621	9.2	113120	16.3	
Urbanization level					<0.001
Urban	8117	28.4	214843	31.0	
Suburban	12278	43	297038	42.9	
Rural	8156	28.6	180501	26.1	
Dementia	127	0.4	944	0.1	<0.001

We then performed the multivariate Cox regression model to evaluate the adjusted HRs of dementia. After controlling for age, gender, urbanization level, SES, diabetes, hyperlipidemia coronary artery disease, history of alcohol intoxication, ischemic stroke, intracranial hemorrhage and CCI, patients with mTBI still had high HR. (3.26; 95% CI, 2.69–3.94) Other independent risk factors of dementia included females, older age, diabetes, higher CCI, ischemic stroke, intracranial hemorrhage, lower SES and living outside of urban area. The statistical results are summarized in Table 2.

**Table 2:** Adjusted Hazard Ratios (HR) of Dementia Because of mTBI.

Variables	Hazard ratio	95% confidence interval	P-value
mTBI	3.26	2.69–3.94	<0.001
Male	0.65	0.57–0.73	<0.001
Patient age	1.12	1.11–1.12	<0.001
Charlson Comorbidity Index Score			
0	1	–	–
1	1.29	1.06–1.56	0.01
≥2	1.47	1.21–1.79	<0.001
Diabetes	1.47	1.27–1.69	<0.001
Hypertension	1.06	0.92–1.22	0.445
Coronary artery disease	1.00	0.87–1.15	0.995
Hyperlipidemia	0.90	0.78–1.04	0.157
History of alcohol intoxication	1.12	0.53–2.36	0.766
Ischemic stroke	2.05	1.43–2.94	<0.001
Intracranial hemorrhage	1.43	1.24–1.66	<0.001
Socioeconomic status			
Low	1	–	–
Moderate	0.73	0.62–0.84	<0.001
High	0.26	0.13–0.52	0.002
Urbanization level			
Urban	1	–	–
Suburban	1.22	1.04–1.42	0.014
Rural	1.22	1.02–1.45	0.027

A subgroup analysis based on patients older than 65 years was performed. There were 6028 patients in the mTBI group and 84392 in the control group. After controlling for the same covariates, the risk in patients with mTBI is still significant. (HR 3.27; 95% CI, 2.67–4.00). The statistical results are similar with the primary study cohort and are summarized in Table 3.

**Table 3:** Adjusted Hazard Ratios (HR) of Dementia Because of mTBI in Patients Older than 65 Years.

Variables	Hazard ratio	95% confidence interval	P-value
mTBI	3.27	2.67–4.00	<0.001
Male	0.62	0.54–0.71	<0.001
Patient age	1.08	1.07–1.09	<0.001
Charlson Comorbidity Index Score			
0	1	–	–
1	1.26	1.02–1.56	0.031
≥2	1.43	1.16–1.77	0.001
Diabetes	1.36	1.17–1.58	<0.001
Hypertension	0.99	0.85–1.14	0.839
Coronary artery disease	1.04	0.90–1.21	0.559
Hyperlipidemia	0.83	0.71–0.97	0.017
History of alcohol intoxication	1.48	0.70–3.12	0.303
Ischemic stroke	1.47	0.95–2.28	0.084
Intracranial hemorrhage	1.39	1.20–1.62	<0.001
Socioeconomic status			
Low	1	–	–
Moderate	0.79	0.67–0.93	0.005
High	1.05	0.43–2.53	0.922
Urbanization level			
Urban	1	–	–
Suburban	1.17	0.99–1.38	0.070
Rural	1.11	0.92–1.35	0.276

Another analysis focusing on patients admitted with TBI was performed. There were 6138 patients in the hospitalized group and 692382 in the control group. After controlling for the same covariates, we observed a higher HR in the hospitalized group (HR 4.44; 95% CI, 3.23–6.10). (Table 4).

**Table 4:** Adjusted Hazard Ratios (HR) of Dementia in Patients Admitted with TBI.

Variables	Hazard ratio	95% confidence interval	P-value
mTBI	4.44	3.24–6.10	<0.001
Male	0.65	0.57–0.74	<0.001
Patient age	1.12	1.11–1.13	<0.001
Charlson Comorbidity Index Score			
0	1	–	–
1	1.35	1.10–1.65	0.003
≥2	1.57	1.28–1.92	0.001
Diabetes	1.38	1.19–1.60	<0.001
Hypertension	1.02	0.88–1.18	0.819
Coronary artery disease	1.09	0.95–1.26	0.225
Hyperlipidemia	0.94	0.80–1.09	0.403
History of alcohol intoxication	1.58	0.82–3.05	0.175
Ischemic stroke	2.00	1.37–2.94	<0.001
Intracranial hemorrhage	1.42	1.22–1.66	<0.001
Socioeconomic status			
Low	1	–	–
Moderate	0.73	063–0.85	<0.001
High	0.24	0.11–0.50	<0.001
Urbanization level			
Urban	1	–	–
Suburban	1.18	1.00–1.38	0.052
Rural	1.15	0.96–1.38	0.132

**Figure 3:**
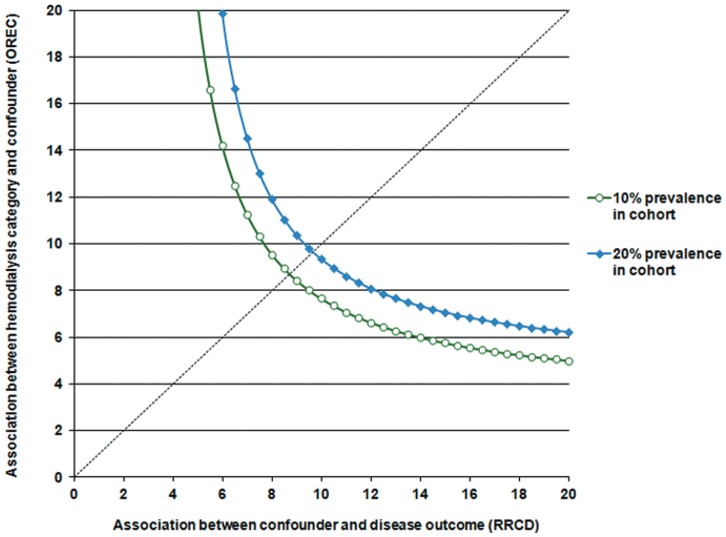
Sensitivity analyses of an unmeasured confounding.

Sensitivity analyses showed that an unmeasured confounder present in 10% of study population would be required to elevate the risk of dementia by a factor of 10 and would also have to have a prevalence among patients with mTBI that would be around 10 times that among the unexposed group to explain a lower 95% confidence limit HR of 2.69. (Figure 3).

### Limitations

First, our findings are generated from administrative data. The primary outcome of dementia was derived from ICD-9-CM code, which is good for insurance reimbursement rather than being the substitute of precise operative definition. The validity of the diagnosis, i.e. sensitivity, specificity and accuracy, cannot be assessed. To overcome this misclassification bias, we restricted our inclusion to patients who were registered in the CIC or those prescribed with medications solely for dementia, as there should be impossible for false positive cases on this side of disease spectrum. Epidemiologic study showed the prevalence of dementia in the elderly (older than 65 years old) in Taiwan ranged from 1.7% to 4.4%, [17] which is similar with our sampled data (1.55%; 2000 cases in a total of 129122 people). The prevalence in 5-year age bands from 65 to 84 years, and for those aged 85 years and older was 0.41%, 0.82%, 1.62%, 2.55% and 4.0%, respectively. Although frequencies were lower compared with the global report published by Cleusa et al, [18] the upward trends were the same. We acknowledged that dementia patients with mild symptoms would not be enrolled and this approach decreases the generalizability of study findings, but we had decided this a must trade-off.

Second, Coding errors are also common in mTBI. [13], [19] In Bazarian et al., [13] the sensitivity of ICD-9-CM codes for mTBI was 45.9% with a specificity of 97.8%. In other words, people in the mTBI group are highly possible to have mTBI, while some people in the unexposed group may still have mTBI during the study period but the inclusion strategy failed to identify them. In this situation, the predicted effect of mTBI on dementia should be toward the null, but we still found significant risk of developing dementia in patients with mTBI.

Third, we could not obtain the clinical information of patients with mTBI, such as the Glasgow Coma Scale, findings on computed tomography of head, the injury mechanism and the initial presentations. By definition, labeling our cases as ‘mTBI’ may be inappropriate. However, it has been validated that the ICD9-CM codes have high specificity regarding diagnosis of mTBI. [13] Furthermore, we excluded those who were admitted to the hospitals to make sure the patients enrolled are really “mild”. Based on our inclusion criteria, although not totally precise, we think the cases in our study group are highly correlated with the definition of mTBI.

Fourth, the average duration from TBI to the diagnosis of dementia is short and we admitted that reverse causation (i.e., undiagnosed dementia resulted in TBI) might also exist. However, we excluded all dementia patients in the first 3 years of our study period to eliminate the effect as possible. Of note, patients in TBI group are older; it is possible the neurodegenerative impact after TBI could have stronger effect on the elderly. Furthermore, we found higher HR of dementia (4.44) in patients admitted with TBI and the reverse causation could not fully explain the ‘dose-response’ of injury severity.

In the last, although we extensively adjust for possible comorbidities, unmeasured cofounding is still an issue. Based on the nature of our dataset, we cannot take some important risk factors of dementia such as gene or family histories into account. However, these risk factors are unlikely associated with mTBI and it is reasonable that these are not confounders in our study. [20] Furthermore, the adjusted HR is significant enough that the residual confounding may not be able to fully explain the result.

## Discussion

Studies have found a history of head trauma was associated with increase in the risk for AD in the absence of a family history of dementia.[3]–[8] In Plassman et al, [8] 548 World War II veterans hospitalized during military service between 1944 and 1945 with a diagnosis of nonpenetrating TBI were compared with 1228 patients matched on education and age. A history of severe and moderate TBI increased the risk of dementia, but there was no significant risk of dementia (HR, 1.33; 95% CI, 0.51–3.47) in those with mTBI.

In our study, we further extend the impact of TBI to the mild type, utilizing the largest cohort study to date to identify that patients with single mTBI have higher risk of developing dementia later in their lives compared to general population. Our study is power enough to provide precise estimate of HR (3.26; 95% CI, 2.69–3.94), which is statistically and clinically significant. The database is well corresponded to the whole population; therefore, loss of follow-up or selection bias were not concerns.

Other findings in our study are consistent with previous publications. First, age is still the single strongest precipitating factor for dementia. [21] Other risk factors such as female, diabetes, stroke (either ischemic or hemorrhagic) and SES were illustrated before.[22]–[25] Of note, hypertension, hyperlipidemia, history of alcohol intoxication and coronary artery disease were not associated with increased risk of dementia in our study. The possible reasons are that there are still conflicting results in publications, [26], [27] and their effects on dementia may be partly explained by the coexisting comorbidities or CCI.

In conclusion, TBI is an independent significant risk factor of developing dementia even in the mild type. The result indicates that more emphasis on the head injury prevention would be worthy.
